# Out-of-Distribution Detection of Human Activity Recognition with Smartwatch Inertial Sensors

**DOI:** 10.3390/s21051669

**Published:** 2021-03-01

**Authors:** Philip Boyer, David Burns, Cari Whyne

**Affiliations:** 1 Orthopaedic Biomechanics Lab, Holland Bone and Joint Program, Sunnybrook Research Institute, Toronto, ON M4N 3M5, Canada; cwhyne@sri.utoronto.ca; 2 Institute of Biomedical Engineering, University of Toronto, Toronto, ON M5S 1A1, Canada; 3 Division of Orthopaedic Surgery, Department of Surgery, University of Toronto, Toronto, ON M5S 1A1, Canada; d.burns@utoronto.ca

**Keywords:** human activity recognition, out of distribution, anomaly detection, open set classification, physiotherapy, inertial sensors, smart watch, rehabilitation, machine learning

## Abstract

Out-of-distribution (OOD) in the context of Human Activity Recognition (HAR) refers to data from activity classes that are not represented in the training data of a Machine Learning (ML) algorithm. OOD data are a challenge to classify accurately for most ML algorithms, especially deep learning models that are prone to overconfident predictions based on in-distribution (IIN) classes. To simulate the OOD problem in physiotherapy, our team collected a new dataset (SPARS9x) consisting of inertial data captured by smartwatches worn by 20 healthy subjects as they performed supervised physiotherapy exercises (IIN), followed by a minimum 3 h of data captured for each subject as they engaged in unrelated and unstructured activities (OOD). In this paper, we experiment with three traditional algorithms for OOD-detection using engineered statistical features, deep learning-generated features, and several popular deep learning approaches on SPARS9x and two other publicly-available human activity datasets (MHEALTH and SPARS). We demonstrate that, while deep learning algorithms perform better than simple traditional algorithms such as KNN with engineered features for in-distribution classification, traditional algorithms outperform deep learning approaches for OOD detection for these HAR time series datasets.

## 1. Introduction

Human activity recognition (HAR) constitutes automatic characterization of activity and movement through intelligent “learning” algorithms. Under the HAR umbrella lies the detection and identification of such varied tasks as hand gestures [1], walking up stairs [2], commuting [3], detecting falls [4], and even smoking [5], with applications in a growing number of fields, including physiotherapy [6]. HAR can be accomplished via a variety of strategies, including machine vision technology [7,8,9] or analysis of inertial data. Inertial measurement units (IMUs) are embedded in many widely available commercial devices, including smart phones [10,11], and smartwatches [6,12]. IMUs enable data capture in natural (home or varied) settings without any need to perform actions in front of a fixed camera or other apparatus. The analysis of inertial data via machine learning (ML) has been demonstrated to yield robust HAR [6].

In attempting to track specific human activities in unsupervised environments, subjects may perform unexpected, unknown, or unrelated activities. This presents a challenge to the use of ML based classifiers as training a ML algorithm on all possible human actions for activity recognition is impractical, and supervised ML algorithms may not accurately classify such out-of-distribution (OOD) activities. In the context of identifying at-home physiotherapy exercises with a smartwatch, subjects may be instructed to only wear a watch while performing their exercises. However, even in this case, subjects may perform other activities between exercises—such as getting a drink of water—or may forget to remove their smartwatch entirely after completing their exercise routines and go about other daily activities. A classification process for at-home physiotherapy exercises that does not include OOD detection risks detecting these activities as exercises, and thereby reporting incorrect estimations of physiotherapy participation and adherence. 

Methods to address the OOD problem exist for image classification but have been less widely applied in the context of time series activity recognition. In this paper, we experiment with several methods commonly used in the image domain to address the OOD problem in the context of shoulder physiotherapy activity recognition. We test three traditional algorithms for OOD-detection on engineered statistical features (One-Class State Vector Machine (OCSVM) [13,14], K-Nearest Neighbor (KNN) [15], and Kmeans [16]), KNN with deep feature embeddings generated by two neural network models, and well-known methods in the image domain based on deep learning: thresholding Softmax confidence [17], confidence calibration via entropy regularization [18], confidence calibration via temperature scaling and input perturbations (ODIN) [19], and extending the Softmax layer to allow prediction of an unknown class (OpenMax) [20]. We evaluate these techniques on a novel physiotherapy exercise dataset we collected (SPARS9x) as well as on two publicly-available activity datasets (MHEALTH [21] and SPARS [6]). 

Contributions of this study include:

- A new physiotherapy activity dataset SPARS9x (DOI: 10.21227/cx5v-vw46), with additional inertial data captured from the smartwatch of each subject while they performed activities of daily living. We believe this study is unique in its approach of capturing a dataset that explicitly simulates the distinction between known target human activities and unknown a priori OOD activities.
- Evaluation of methods of OOD detection from the image domain as applied to physiotherapy inertial data captured by smartwatches, in comparison to traditional algorithms using both hand-crafted engineered statistical features and deep learning model-derived features.

This paper is organized as follows. In Section 2, we include a brief synopsis of OOD detection methods and other background important to this study. Section 3 provides an overview of the OOD detection methods that are explored and the datasets used as well as details related to the setup of the analyses, including model architectures and evaluation methods used. Section 4 presents the results of the analyses and Section 5 the discussion. Section 6 proposes potential future work, Section 7 details the study limitations, and Section 8 provides a summary and concluding remarks. 

## 2. Background and Related Work

### 2.1. Human Activity Recognition with Machine Learning of Inertial Data

There is a large body of research on the use of IMUs and the inertial data they collect for HAR [22,23,24], with a considerable subset evaluating the practical applications of these technologies in the health domain [25]. In particular, gait assessment [26,27] and fall prediction [28,29] have garnered significant attention, likely due in large part to their immediate clinical need. More recently, researchers have also begun applying these same methodologies to physiotherapy and rehabilitation [6,30,31,32] where unique challenges may be encountered. The focus of these studies has typically been the exploration of techniques for improving the accuracy of correctly identifying physiotherapy exercises. Less effort has been spent on identifying when subjects are performing the exercises that are within the scope of activities represented in the training dataset. 

In a closed-set classification problem, an ML model is tasked with predicting the class that a sample belongs to from within a set of classes that the ML model was trained on. However, input samples may deviate from these classes, often into classes that are both previously unknown and unforeseen. The challenge of accurately identifying such samples is known as the out-of-distribution (OOD) or open set classification problem (for a review of OOD detection methodologies, see [33]). It is worth noting that OOD detection is often conflated with novelty, anomaly, or outlier detection in the literature [34]. However, predictions in the case of novelty or outlier detection in time series data often implies identifying deviation from an expected input based on a chosen error metric [35,36,37,38,39,40].

OOD detection methods are commonly employed in the image domain, but many of these solutions have largely remained untested in the HAR time series domain. The following sections provide a short summary of OOD detection methods of interest to this study. 

### 2.2. Out-of-Distribution Detection Techniques

Scheirer et al. formalized the definition of the open set classification problem and identified a binary SVM method to separate open set inputs from the closed set with a linear kernel [41]. This method was then extended by Scheirer et al. with Compact Ablating Probability (CAP) to identify unknown class samples and Extreme Value Theory (EVT) to calibrate prediction results using a Weibull distribution [42]. Many techniques have been proposed for OOD detection based on traditional classification algorithms, such as OCSVM [41,43,44,45], KNN [15], and Kmeans [16]. Recently, deep learning-based OOD-detection techniques have been proposed [17,19,20,26,27,28,29,30,31,32,33,34,35,46,47,48,49,50,51,52,53,54,55], some of which we now highlight.

In deep neural network classifiers with multi-class outputs, the final layer commonly performs a Softmax operation on the output from the previous layers (the “core”), which transforms input values into probabilities estimates between 0 and 1, the total of which sum to 1. The Softmax layer is typically used in deep learning classifier models to assign probability estimates or activations for input samples to known classes. One weakness of the Softmax layer is that all predictions must be assigned to one of a range of predetermined classes. To detect OOD samples, the activation, or “confidence”, of the Softmax prediction may be evaluated against a threshold, whereby input data that elicit a maximum Softmax output below the threshold may be designated as out-of-distribution [17]. However, Softmax outputs are prone to overconfident predictions, which may impact the accuracy of this approach [56]. Other methods have been proposed to ameliorate the overconfidence issue, including temperature scaling [19,56] and entropy regularization [18]. Alternatively, one may reformulate the Softmax layer to predict an unknown class, such as with the OpenMax technique [20]. Traditional and deep learning algorithms may be used together, such as using an OCSVM to detect anomalous motion (e.g., jumping and walking) with features extracted from accelerometer and gyroscopic data by a convolutional neural network [57].

Generative adversarial networks (GANs) have also been explored for the OOD problem [58]. GANs are generally incorporated into an open set discriminative framework by training a GAN on the known input dataset and converting the trained discriminator into an OOD classifier [46,59]. Alternatively, the generator used to synthesize samples may designate them as from an OOD class, and then add them to the full training set used to train a classifier [47,48,49,50]. However, such GAN augmentation techniques may produce samples that do not adequately cover the entirety of in-distribution (IIN) decision boundaries, resulting in non-optimal OOD classifiers [60]. Searching a trained generator’s latent space for a close match to an input is another approach [61]. Autoencoders or similar compact representation models may also be used to detect OOD-samples based on high input reconstruction error [51,52,53,54], or in adversarial frameworks with clustering methods [55]. Recently, generative likelihood ratio methods for OOD detection have been proposed, including that of Ren et al. for genomics data with application to the image domain [62].

### 2.3. OOD Detection with Inertial Data

Studies on OOD detection in the context of HAR inertial data are less common than those in the image domain. In the recent HAR time series outlier-detection work by Organero, a model is trained on a particular activity for each user and the Pearson correlation coefficient is used to identify deviation of real data from predicted data [36]. Omae et al. deployed a convolutional neural network to perform feature extraction of accelerometer and gyroscopic data from the swimming activity, and an OCSVM is used to detect anomalous motion (e.g., jumping and walking) based on extracted features in consideration of an individualized optimization algorithm [57]. It is notable that each of these studies either approach the problem in terms of anomaly detection, and/or use “traditional” algorithms such as OCSVM to identify OOD data. To our knowledge, the selection of solutions in the image domain for OOD detection presented in this study (i.e., Softmax Thresholding [17], ODIN [19], and OpenMax [20]) have not yet been applied to the domain of HAR time series inertial data, nor has a direct comparison of these to methodologies using traditional algorithms using engineered or deep learning features been performed. 

## 3. Materials and Methods

### 3.1. Out-of-Distribution Detection

In this section, an overview of methods considered for OOD detection on HAR time series datasets is presented. The deep learning approaches are well-known in the image domain.

#### 3.1.1. One Class State Vector Machines (OCSVMs)

One class state vector machines (OCSVMs) test whether an input sample is a member of a single class of interest. For the OOD problem, this one class would correspond to all classes of IIN data. Using the approach described by Schölkopf et al. [13], data are cordoned off from the origin with a hyperplane, and data beyond this plane are classified as OOD. 

#### 3.1.2. K-Nearest Neighbor (KNN)

The K-Nearest Neighbor (KNN) algorithm finds the (Euclidean) distance between inputs. For classification, KNN typically uses a voting mechanism based on the closest N-neighbors to the location in space of an input to be tested. OOD classes can be identified when the distance to the closest N-neighbors exceeds a given threshold. 

#### 3.1.3. Kmeans

Kmeans is an unsupervised algorithm that uses an iterative updating process to assign similar data to clusters. OOD data can be detected by comparing the distance to the center of the cluster assigned to an input against a threshold value.

#### 3.1.4. Deep Feature Embedding

Deep features refer to the outputs of layers immediately preceding the dense/Softmax layers of fully trained deep neural network models, effectively embedding test data into the feature space of each core model. This deep feature representation may be used with traditional algorithms that require vectorized input data (e.g., KNN). We use an L2 normalization layer in our deep models to produce an embedding with consistent scale in the feature space. 

#### 3.1.5. Softmax Thresholding

It is common practice in deep learning classification algorithms to base predictions on the maximum output of a Softmax layer [17]. The output of the Softmax layer is often interpreted as the confidence of a model in its prediction that the sample is from a particular class [56]. If the Softmax output is used as a confidence metric, samples that fail to meet that threshold may be classified as OOD.

#### 3.1.6. Entropy Regularization

Softmax predictions in deep learning models are prone to overconfidence [63] and may be adjusted through confidence calibration techniques. Entropy-regularization accomplishes this by penalizing the negative log-loss with the addition of a negative entropy term that effectively regularizes the loss to prevent overconfident predictions. This has been shown to be an effective technique on several diverse datasets with models incorporating dense, convolutional (CNN), and long-short term memory (LSTM) layers [18]. The intuition behind this technique for OOD detection is that reducing overconfidence may allow for better discrimination of the more confident Softmax predictions of IIN activities from the less confident predictions of OOD activities. 

#### 3.1.7. ODIN

The out-of-Distribution detector for neural networks (ODIN) is a two-part method for OOD detection, performed post-process during prediction by a trained network [19]. In temperature scaling, the logits (the output of the final output layer of a model) of the classifier are scaled by a scalar value called the “temperature” (T) prior to performing Softmax. 

The input data are then perturbed by a fixed amount, which has a greater effect on the IIN data than the OOD data, increasing their separation. The input perturbations are based on the directionality of input gradients, obtained by backpropagating the inputs through the network once. The absolute magnitudes of the inputs are perturbed by an epsilon value opposite the gradient direction. The perturbed and temperature-scaled inputs are run through the classifier, and OOD samples are detected as those falling beneath a learned threshold of Softmax. 

#### 3.1.8. OpenMax

OpenMax [20] is a model layer that assigns a probability to an unknown class. It is a replacement for the Softmax layer. Its core concept is to reject inputs that are “far” from the training data distribution in feature space. Each class is represented as a point with the mean calculated from the correctly classified training examples. A Weibull distribution is fitted on the largest distances between all correctly identified training samples of a class and the mean. The result is a probability that an input is from this class, and a set threshold is used to determine OOD. 

Unlike Softmax, the OpenMax layer does not restrict probabilities to sum to 1. Instead, a pseudo-activation (of the logits, also termed the activation vector) is calculated for an OOD class by using the Weibull distribution to redistribute activations to include an unknown class. This can be used to identify OOD data if this class has the highest probability output from the OpenMax layer.

### 3.2. Experimental Setup

#### 3.2.1. Experimental Datasets

This study considers two publicly available inertial sensor datasets, MHEALTH (Mobile Health) [21] and SPAR (Supervised Physiotherapy Activity Recognition) [6], and a novel dataset (SPARS9x) we collected specifically to assess OOD detection. The properties of these datasets are summarized in Table 1. Only the data from inertial devices attached to one wrist from each study subject in MHEALTH are used, effectively simulating smartwatch IMUs.

The new inertial dataset, SPARS9x (Smart Physiotherapy Activity Recognition System 9-axis), was captured from 20 healthy subjects (8 male, 12 female, median age 25). Supervised physiotherapy exercises, followed by a minimum 3 h of data from unrelated and unstructured activities (OOD data), were captured from a smartwatch with a 9-axis IMU (accelerometer, gyroscope and magnetometer). This study was approved by the Research Ethics Board of Sunnybrook Health Sciences Centre.

**Figure 1:**
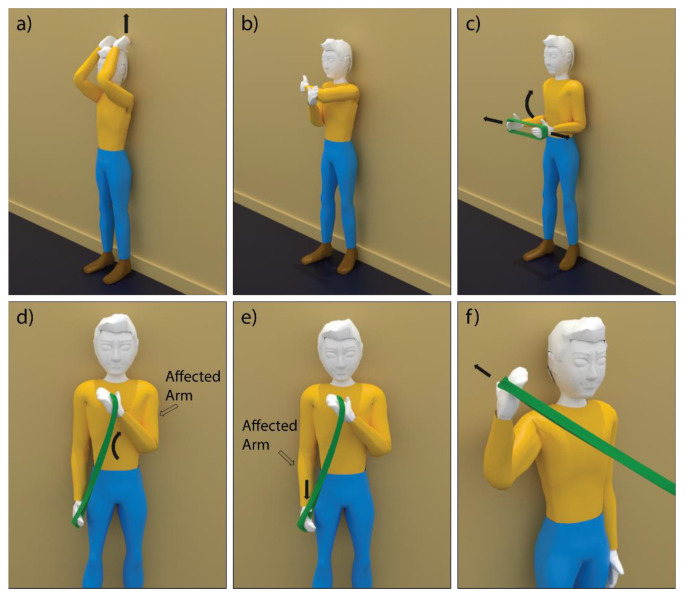
SPARS9x concentric exercises: (**a**) active flexion; (**b**) cross chest adduction; (**c**) shoulder girdle stabilization with elevation; (**d**) biceps muscle strengthening; (**e**) triceps pull downs; and (**f**) external rotation in 90-degree abduction in the scapular plane. Black arrows indicate direction of motion or tension.

Shoulder physiotherapy exercises in SPARS9x were selected from the Basic Shoulder Rehabilitation Program provided by the Sunnybrook Holland Orthopaedic & Arthritic Centre. Exercises were selected to include both concentric (muscle activation with shortening – shown in Figure 1) and isometric (muscle activation without length change) types. Exercises were performed with a resistance band when needed. Concentric exercises included: active flexion, cross chest adduction, shoulder girdle stabilization with elevation, biceps muscle strengthening, triceps pull downs, and external rotation in 90-degree abduction in the scapular plane. Isometric exercises included external rotation, internal rotation, abduction, and extension.

#### 3.2.2. Data Transformation Pipeline

The raw time series inertial data were split into equally-sized windows, with constant step size of 1 s, using the Seglearn package for time series machine learning [64]. Samples counts at 10s window size for each dataset were 24,004 for SPARS9x, 9527 for SPARS, and 5676 for MHealth. Artifacts in SPARS were located and removed based on sampling rate and duplicate time stamp checks. For SPARS9x, each record was plotted and visually inspected for artifacts immediately after each subject’s session, and records that contained artifacts were either truncated or sessions were re-recorded.

Inputs to traditional models are often generated based on statistics describing the raw data, such as the mean and kurtosis of each input channel [1]. The statistical features engineered for input to the traditional algorithms in this analysis are median, absolute energy (root mean squared), standard deviation, variance, minimum, maximum, skewness, kurtosis, mean spectral energy, and mean crossings. These are also generated by the Seglearn Python package.

For the purposes of this study, we define OOD activities as any activity performed by a subject that is not in the list of labeled activities available to a classifier during training. The activities performed in SPAR [6] and MHEALTH [21] are described elsewhere. However, it is especially important to note which classes of these datasets are to be designated as OOD for reproducibility of these experiments since activities similar to IIN classes may be more difficult to detect as OOD. Highly confused classes were those identified through in-distribution classification, corresponding to activities of similar motion that produce patterns in the data that are difficult to discriminate between by machine learning classifiers (e.g., jogging and running in MHEALTH). In general, highly confused classes in supervised classification were kept together in IIN for these experiments. Beyond that, OOD classes were simply selected as the final two and three activities in the order of activities for SPARS and MHEALTH respectively. Lower trapezius row with resistance band and bent over row with 3 lb. dumbbell are selected as OOD classes for SPARS. Frontal elevation of arms, cycling, and jump front and back are selected as OOD activities for MHEALTH. MHEALTH null class (Class 0) is not used for this analysis as it is unclear how much overlap there is between those data and the in-distribution activities (e.g., walking or standing), potentially skewing results. Only concentric exercises from SPARS9x are used for this analysis. 

Since the recording time of OOD data for each subject significantly exceeds that of their physiotherapy exercises, for this study, the length of each OOD record in SPARS9x was cut to match the length of time spent performing target exercises. Magnetometer data are excluded for these experiments from SPARS9x.

#### 3.2.3. Model Architecture

Two separate deep neural networks were tested as core architectures in the analysis. 

The first core is a convolutional recurrent neural network (CRNN), which was found in our previous study to perform well on classification tasks [6]. In the particular case of time series data, temporal relationships may be captured by recurrent neural network (RNN) layers [65]. This model consists of two convolutional layers with 128 filters and kernel size of 2, followed by RELU and Maxpooling layers. Next, the outputs of the convolutional layers are passed into two LSTM layers of 100 units each and dropout of 0.1. This is consistent with findings in literature that suggest that two sequential LSTM layers may be beneficial when processing time series data [66].

The second core is a fully convolutional neural network (FCN) used for time series classification [67], which we have previously shown to be effective in a personalized activity recognition approach [68]. 

A final dense layer outputs the logits for each class (prior to Softmax, method depending). Hyperparameters of each core were grid-searched for their optimal values in our previous works [6]. The deep learning models were developed in PyTorch. The traditional models were implemented using packages from the scikit-learn Python library.

#### 3.2.4. In-Distribution Classification Experiments

Supervised classification of in-distribution activities is performed to validate the performance of the core models with each of the three datasets.

We compare performance of:

- KNN with engineered statistical features
- KNN with deep features (CRNN/FCN)
- CRNN and FCN Cores

#### 3.2.5. Out-of-Distribution Prediction Experiments

We compare the following methods for OOD detection:

- Traditional algorithms: KNN, OCSVM, and Kmeans with engineered features
- KNN with deep features (CRNN/FCN)
- Deep learning methods: SoftMax threshold, entropy regularization, ODIN, and OpenMax

#### 3.2.6. Class Removal Experiments

The lowest confidence classes (or those with the lowest mean activations or greatest overlap with OOD) in the OOD detection experiments according to SoftMax prediction confidence (i.e., potentially the most likely to be confused with OOD classes) are identified in the SPARS9x and MHEALTH datasets and either removed from the experiments (SPARS9x) or moved into the IIN training set (MHEALTH). OOD experiments are then repeated for these two datasets at a segment length of 10s to evaluate impact on OOD detection. These experiments are performed to analyze the effect that removing or re-designating highly confused classes (in terms of OOD detection) has on OOD prediction performance.

#### 3.2.7. Training and Validation

A grid search is performed on segment length for each experiment, with segment sizes from 2 to 10 s at 2 s intervals. A 5-fold cross-validation strategy is used with equal-sized folds for each experiment. Each deep learning model uses a batch size of 256 and is run for 100 epochs. Testing was performed on a system with an NVidia GTX1080 GPU with 8 GB on-board memory, an Intel Core i7-6700 CPU (@3.4 GHz), and 16.0 GB of RAM.

#### 3.2.8. Evaluation Metrics

For the in-distribution classification tasks, an accuracy metric was used, as these datasets demonstrated approximate equal class balance. In the OOD problem, we are concerned with how well a method is able to differentiate between distributions, which can be measured by the area under the receiver operating curve (AUROC) metric. The AUROC can be interpreted as a measure of how separable the IIN and OOD data are under the model.

## 4. Results

### 4.1. In-Distribution Classification

Table 2 presents the in-distribution supervised classification experiment results. Deep learning methods or KNN with deep features consistently outperformed KNN with engineered features. 

**Figure 2:**
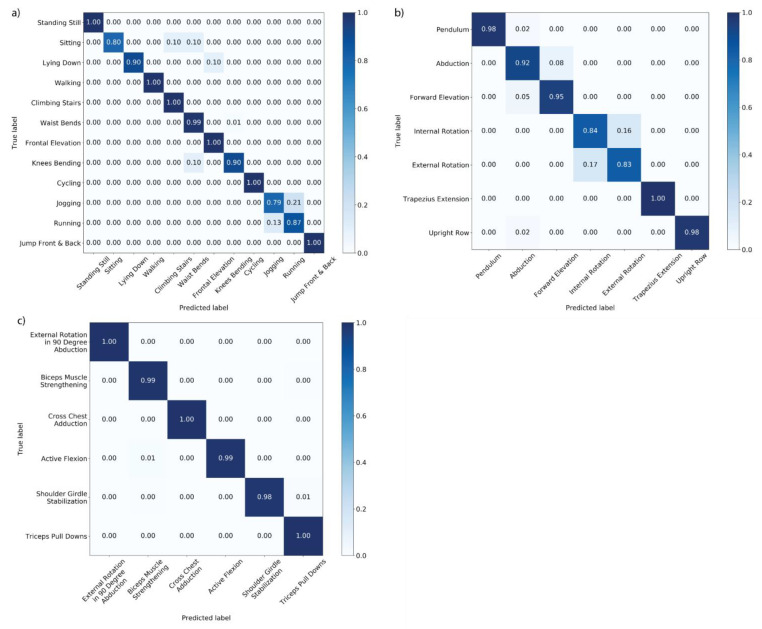
In-distribution classification confusion matrices with KNN with FCN Deep Features at 10 s segment size for: (**a**) MHEALTH; (**b**) SPARS; and (**c**) SPARS9x.

Figure 2 shows the confusion matrices for each of the three datasets for classification using a KNN with deep learning features as input generated by the FCN architecture at 10s segment size. As reflected in Table 2, the KNN algorithm is able to classify SPARS9x with near perfect accuracy, whereas there are pairs of highly confused classes for both SPARS (Internal Rotation/External Rotation) and MHEALTH (Jogging/Running). 

### 4.2. Out-of-Distribution Detection

Table 3 presents the results of the OOD experiments. Each reported accuracy results includes the standard error of five folds for the grid search of optimal hyperparameter settings.

Traditional algorithms performed better than the deep learning-based models. The KNN method in particular achieved superior results. Among the deep learning algorithms, Softmax thresholding without any other intervention was particularly ineffective, while OpenMax generally performed best. Deep feature embedding with KNN yielded competitive results compared to the deep learning methods. 

**Figure 3:**
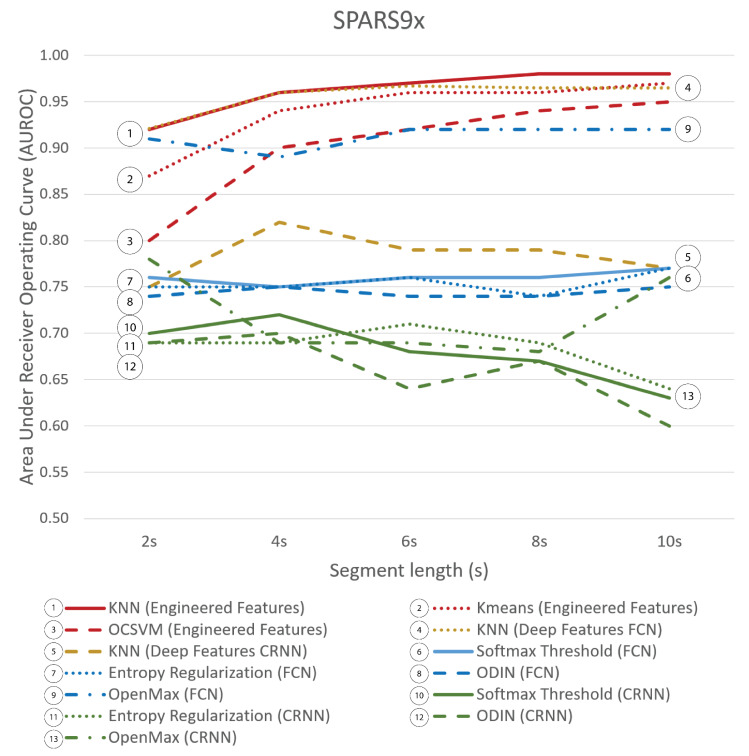
OOD-Detection AUROC for SPARS9x for each method by segment length. Traditional algorithms with engineered features are shown in red (1–3), deep features in yellow (4 and 5), deep learning approaches with FCN in blue (6–9), and deep learning approaches with CRNN in green (10–13).

Figure 3 illustrates the results of a segment length grid search for each of the algorithms on SPARS9x. Increasing segment length consistently improved accuracy for the traditional algorithms but not for the deep learning algorithms. 

### 4.3. Class Removal Experiments

**Figure 4:**
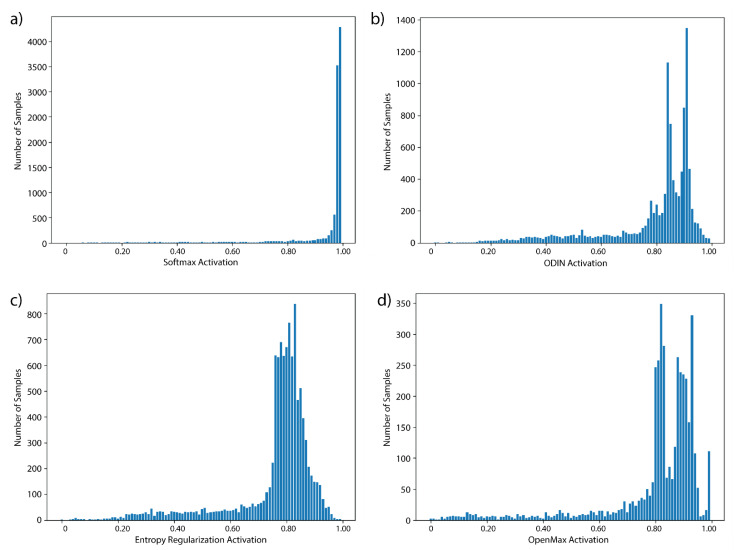
Distributions of activations of SPARS9x prediction with CRNN core: (**a**) Softmax activation; and (**b**–**d**) activation post-processing with ODIN, entropy regularization, and OpenMax respectively. Activations are scaled to between 0 and 1 for each method for illustrative purposes.

The distributions of activations for the four deep learning algorithms for SPARS9x with the CRNN core are shown in Figure 4. Distributions in vanilla Softmax in Figure 4a are clustered near to the maximum of 1.0, whereas the other algorithms appear to be effective at reducing overconfidence judging by their improved spread. Mean activations with the FCN core of the four deep learning algorithms for each activity of SPARS9x are shown in Figure 4. This figure illustrates a similar reduction in overconfidence for the FCN, but it is important to note that there is still significant overlap in activations between the OOD and IIN classes for each. 

**Figure 5:**
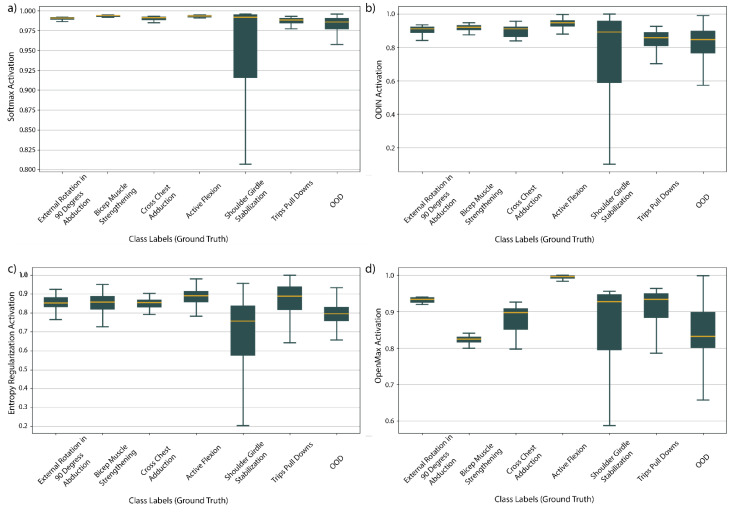
Mean activations for each method by ground truth class labels for SPARS9x prediction with FCN core: (**a**) Softmax activation; and (**b**–**d**) activation post-processing with ODIN, entropy regularization, and OpenMax respectively. Activations are scaled to between 0 and 1 for each method for illustrative purposes.

The boxplots in Figure 5a demonstrate the lower range of mean Softmax activations for the shoulder girdle stabilization activity with the FCN model for SPARS9x. Removal of this activity class from the experiments results in less overlap of Softmax confidence with the OOD data, but also results in altering the confidence in predicting every remaining class. 

**Figure 6:**
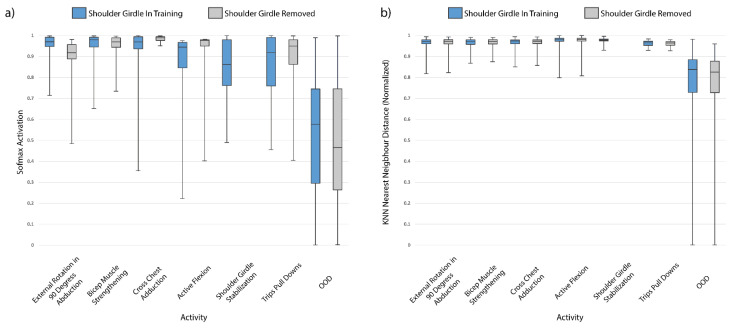
Effect on shoulder girdle stabilization exercise inclusion/exclusion from training data for OOD detection of the SPARS9x dataset on: (**a**) FCN Softmax activation; and (**b**) normalized KNN nearest neighbor distance. This figure highlights the resiliency to changes in training class inclusion/exclusion of the KNN algorithm compared to deep learning algorithms for detecting OOD data with this dataset. Note that, even though the mean Softmax activation decreases for the OOD data and increases for the in-distribution classes with shoulder girdle stabilization removed, there is still a slight decrease in AUROC (see Softmax Threshold of Figure 7b).

**Figure 7:**
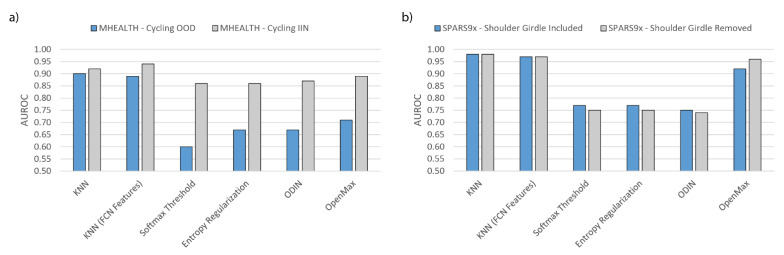
Effect on OOD Detection AUROC of selected algorithms by: (**a**) moving MHEALTH “cycling” class from OOD to IIN; and (**b**) removing the “Shoulder Girdle Stabilization” exercise from SPARS9x. FCN core is used for the deep learning methods.

The KNN nearest neighbor distance predictions for SPARS9x are shown in Figure 6b, where the OOD class samples would appear to already be easily separable from the IIN samples with an appropriately chosen distance threshold. Indeed, it appears that not much is gained in terms of separability with removal of the Shoulder Girdle Stabilization class, and this is reflected in the results shown in Figure 7b, where AUROC of the KNN algorithm remains stable. 

**Figure 8:**
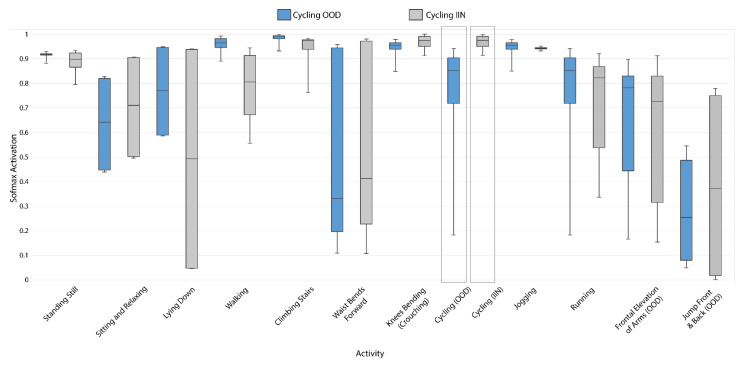
Effect on FCN Softmax activation for each activity in MHEALTH when cycling activity is in OOD and when cycling activity is IIN.

Figure 8 illustrates the effect on Softmax activation from re-designating the cycling activity of MHealth from OOD to IIN. As expected, there is a significant increase in the prediction confidence for cycling (because there are now samples of this activity in the training data), but the important takeaway from this figure is that the activations of each of the other activities are affected as well. 

The effects of class removal or re-designation on each dataset for a selection of methods are shown in Figure 7. Figure 7a illustrates that moving the cycling activity of MHEALTH from OOD to IIN improves OOD detection for each algorithm, especially in the case of the deep learning algorithms. KNN using FCN features for cycling IIN now performs better than the KNN algorithm using engineered features. For SPARS9x, removal of the shoulder girdle stabilization activity, as shown in Figure 7b, yields little improvement, and the KNN algorithm with engineered statistical features still performs better than the other algorithms. 

### 4.4. Train and Prediction Time

Mean prediction times of the models are shown in Table 4. Softmax thresholding and entropy regularization had the shortest prediction times. OpenMax was found to require the longest computation times for OOD prediction. The training times of the base models shown in Table 5 illustrate the longer training times required for deep learning methods.

## 5. Discussion

In this paper, we address the OOD problem in the context of shoulder physiotherapy activity recognition using traditional algorithms on engineered statistical features, deep feature embeddings generated by two neural network models, as well as deep learning approaches. We evaluated these techniques on a novel physiotherapy exercise dataset (SPARS9x) that may best reflect the clinical use case for OOD of physiotherapy data as well as on two publicly-available activity datasets (MHEALTH [21] and SPARS [6]). Deep learning performance was superior to traditional algorithms (i.e., KNN) with engineered features for in-distribution classification; however, surprisingly, the opposite is true for OOD detection of SPARS9x in particular. Since the KNN algorithm also performs worse with deep features for SPARS9x, this would suggest the answer lies in the features generated by the deep learning models themselves. This may be an indication that the deep learning models are learning representations of the data that do not generalize as well to data distributions outside of their training experience for human activity inertial datasets such as SPARS9x. These deep learning models may be learning complex relationships in the data in order to discriminate between in-distribution classes. Due to the inscrutable nature of the models and even the inertial data themselves, it is difficult to describe or understand exactly what the learned deep features represent. In future work, it would be very interesting to identify what aspects of the data the neural network models in this study are using to discriminate classes, and why these are not as effective as using simple engineered features for OOD detection with a dataset such as SPARS9x that explicitly simulates OOD activity data.

HAR classification research involving IMU sensors embedded in wearables is fairly common given the ubiquity of these devices, including those using smartphones [2,10,22,69,70] and smartwatches [5,6,12,71]. Studies examining feature extraction techniques have previously been performed for time series [72,73,74]. However, in this paper, we are interested in the impact of engineered versus deep learning-generated features on HAR inertial data for OOD detection in particular. Numerous human activity inertial datasets exist, with notable examples including MHEALTH [21], PAMAP2 [75], and the recent WISDM gesture-recognition dataset [76]. While these datasets often have an intermediary “null” class between exercises (e.g., MHEALTH), this is often restricted. For example, in the gesture recognition study by Bulling et al. [1], participants were asked not to engage in any other activities between target activities. Classification of repetitive exercises, as in the case of physiotherapy inertial data, add an additional nuance to the analysis. OOD activities may resemble IIN activities, but IIN activities may be distinct in their repetitive nature (e.g., reaching for a mug versus lifting an arm to perform a physiotherapy exercise for several repetitions in a short time frame). We believe this study is unique in its approach of capturing a dataset that explicitly simulates the distinction between known target human activities and unknown a priori OOD activities.

Table 4 where samples in the test set are bucketed according to activation for SPARS9x with a CRNN core. The other deep learning methods, ODIN, entropy regularization, and OpenMax, appear to be effective at reducing this overconfidence for HAR inertial data, judging by the improved spread in the distributions. However, when using confidence to threshold between IIN and OOD classes, relative confidence between classes is more important than absolute confidence. The activations in Figure 5 have been normalized to illustrate this point. Of particular interest in this figure is that some degree of overlap is present for each of the algorithms between the OOD classes and the IIN activities. For every model other than OpenMax (which does not use this information directly for OOD class prediction), this means that thresholding based on activation will be little more accurate than thresholding Softmax, an observation that is borne out in the results of Table 3.

As shown in Figure 7, moving the cycling class of MHEALTH from OOD to INN dramatically increases OOD detection accuracy for deep learning approaches. The algorithms are able to accurately discriminate this class in supervised IIN classification with near perfect accuracy, just as with the other two chosen OOD classes, however, the cycling class in particular is wrongly overconfident. Moving a class that causes confusion as measured by Softmax confidence increases AUROC for Softmax Thresholding, but it also increases KNN prediction AUROC. This is seen as designating the cycling activity as IIN increased the AUROC of Softmax Thresholding from 0.60 to 0.86, but also increased KNN from 0.90 to 0.92. Conversely, in Figure 7b, the KNN algorithm has no such issue with the shoulder girdle stabilization activity, so the advantage that the traditional algorithms have over Softmax cannot be explained by class confusion alone. 

Removing or adding a class to a deep learning model’s training set impacts both the difficulty of the OOD task and also alters the learned feature representation. As shown in Figure 6a, removing a low-confidence class did not greatly improve OOD prediction accuracy. Removing this class alters the model’s parameters which may have decreased generalizability to the test and OOD set. With the case of shoulder girdle stabilization of SPARS9x and cycling in MHEALTH, other classes may become less or more confident as a result. This may explain why removing the shoulder girdle stabilization activity from SPARS9x did not result in a dramatic change as in the case of moving the cycling class to IIN in the MHEALTH dataset.

This is illustrative of some major pitfalls in using activation threshold-based techniques for OOD-detection, including entropy regularization and ODIN: some classes may have more variance than others in how they are performed, and in-distribution classes may share similarities to one another. Either of these issues would reduce confidence in predicting these classes. This spread of confidence explains why Softmax thresholding without any other intervention was found to be a particularly ineffective method, as there is activation overlap between samples from less confident in-distribution classes and OOD samples. This is in contrast to the traditional algorithms such as KNN that did not exhibit this overlap—while discriminating between in-distribution classes were generally less accurate than deep learning methods, the activation (i.e., distance) spreads were still far removed from those of the OOD data. Further investigation reveals that while each of the deep learning methods appear effective at reducing Softmax overconfidence (i.e., entropy regularization, ODIN, and OpenMax), the relative overlap of confidence spread between the IIN and OOD classes appears to differ little from vanilla Softmax. For every model other than OpenMax (which does not use this information directly for OOD class prediction), this means that thresholding based on activation will be little more accurate than thresholding Softmax, an observation that is borne out in the results of Table 3. 

Table 3 demonstrates that choice of segment length may cause large variance in AUROC. This is another advantage that traditional algorithms have over deep learning methods in these experiments—one can set a relatively high segment length of 10s and be confident that this is a reasonable choice that will result in near-optimal prediction accuracy. For deep learning algorithms, optimization of segment length is important to achieve optimal performance of the classification pipeline, and this likely reflects a tradeoff between the model size and number of training samples versus the amount of information available in each individual segment. 

While several methods exist to perform activity segmentation or windowing for time series data, for this analysis, we focus on the sliding window technique due to the periodicity of physiotherapy exercises. Analyses have been performed on the impact of sliding window size for HAR inertial data [77], including adaptive sliding window segmentation techniques [78,79,80]. To our knowledge, an analysis of impact of window size for OOD-detection with HAR inertial data has yet to be performed.

This analysis evaluated the effect of fixed window size on OOD detection. While setting a fixed window size or including that parameter in a hyperparameter grid search is not optimal, to our knowledge existing adaptive window algorithms that have been proposed for inertial data are not easily extensible to the OOD problem as they rely on first predicting the class the segment belongs to. As an example, the adaptive window size algorithms as proposed by Noor et al. [78] increases window size based on increasing probability that a segment is from a particular predicted activity, activity information which would not be available in the OOD case. While an adaptive sliding window algorithm such as this may help differentiate between IIN activities, it is unclear if it would be beneficial in identifying OOD activities. This point is made clearer when examining the OOD detection in Table 3, where larger window sizes generally increased detection accuracy, versus IIN classification in Table 2, where highest detection accuracies are obtained in the mid-sized windows. This implies that the optimal window size for OOD detection accuracy is dependent on the entirety of the dataset, not just the in-distribution ones. 

While confidence trends are similar overall, the CRNN model exhibited much higher Softmax confidence in its predictions than the FCN model (i.e., exhibiting much more of the overconfidence that neural network models are known for). This may be an indication of why the FCN model performs better in most supervised learning scenarios. 

The ODIN method was developed for OOD detection on image datasets (as were many of the other methods tested here), rather than time series datasets. There are manipulations that make sense in the image domain but may be less applicable in the time domain. For example, while it might be reasonable to flip an image as an augmentation method, performing an activity in reverse is not a valid example of that same activity. This argument also applies to input perturbations of ODIN. In the original study by Liang et al. [19], increasing perturbations improved DenseNet results, but significantly negatively impacted Wide ResNet results, where AUROC decreased from 0.93–0.95 to 0.80–0.85. This suggests that the success of this approach at improving OOD detection depends heavily on the model architecture, but the type of dataset is also likely a factor.

The OpenMax results presented in the work of Bendale and Boult are generated by models trained on ImageNet [20] using AlexNet as a feature extractor [81]. To our knowledge, no such large dataset exists for physiotherapy time series data. In this research, we trained with a significantly smaller dataset in a completely separate domain (i.e., inertial time series data). There are also some conceptual concerns with the OpenMax approach. Specifically, as indicated by the range of OpenMax activations we found, this approach may be impacted in a similar fashion to the other methods by the wide variation in activations for some IIN classes. Since the OOD decision in OpenMax is based on the distance from the mean activation vector, and the Weibull distribution is fit to the largest deviations in the correct training samples per class from each mean activation, this method will still make inaccurate predictions if there is significant activation overlap between IIN and OOD activations. Distance from the MAV may be too simplified of an approach in this case. A different distance measure and/or loss function may be needed, such as the il-loss proposed by Hassen and Chan [82], while retaining the remainder of the unknown class approach of OpenMax. A GAN-augmented approach may also be used to ameliorate these issues, but as noted previously, generated samples are unlikely to fully encapsulate the IIN decision-boundary, so such a classifier is unlikely to be an optimal detector [60]. However, as seen in the work by Ge et al., GAN augmentation may still improve results [47].

Traditional algorithms tended to have the lengthiest prediction time requirements, but OpenMax required the longest time to run. As implemented in these experiments, OpenMax uses a costly sample-by-sample approach in generating the final activation vector. Unlike ODIN and OpenMax, entropy regularization only requires a change to the model loss function compared to standard supervised learning and does not require additional costly processing steps in the prediction phase, shortening its prediction time. The increase in prediction times for MHEALTH versus SPARS for traditional algorithms and OpenMax may be partly explained by the greater number of IIN classes in the MHEALTH dataset. 

## 6. Future Work

Regardless of approach, we suggest that future work should attempt to resolve and explain the apparent decrease in generalizability of deep learning features compared to engineered features towards OOD detection with time series human activity inertial datasets similar to SPARS9x. Identifying patterns in the data that are key to discriminating between in-distribution classes by these deep learning models may assist in this process. 

The present work focuses on purely discriminative methods rather than delving into the myriad of generative options available. These generative models may also be combined with traditional algorithms. An adversarial autoencoder method, such as that presented by Pidhorskyi et al. [83], may also be a promising future approach to explore, or one based on likelihood ratios [62].

This work evaluated IIN classification and OOD detection accuracy using a range of fixed window sizes. While this study found that larger window sizes increased OOD detection accuracy, this may not always be the case. A new algorithm for adaptive sliding window segmentation for this kind of periodic HAR data specifically accounting for the potential for either periodic or non-periodic OOD data would be ideal.

## 7. Limitations

The SPARS9x dataset was captured in laboratory conditions with healthy subjects. We expect that performance of exercises by injured patients in the home setting to exhibit more variance in motion than those present in the SPARS9x dataset.

Only the lower arm sensor in the MHEALTH dataset is used, essentially simulating inertial data captured by a smartwatch. Including data from other sensors in this dataset would likely improve classification and detection accuracy, but we limited the data to align with the proposed clinical solution to tracking shoulder physiotherapy with a smartwatch alone. 

Only concentric exercises from SPARS9x were used in this analysis. Isometric exercises in SPARS9x were found to be too highly confused with OOD data in preliminary classification validation experiments to be viable for use in the OOD detection analysis. 

Magnetometer data were excluded from SPARS9x for these analyses as their inclusion was not found to improve classification accuracy when combined with data from the accelerometer and gyroscope in preliminary experiments.

This paper applies a selection of well-known OOD detection techniques from the image domain to HAR time series inertial datasets. There are many other OOD detection methods in use in the image domain and elsewhere, and those tested in this paper represent only a selection of those most widely cited. However, these results are likely reflective of most discriminative models that base prediction on Softmax activation, whether explicitly or implicitly.

## 8. Conclusions

In this paper, we present a novel physiotherapy inertial dataset captured specifically for the analysis of the out-of-distribution problem in HAR and tested a range of OOD detection methods common in the image domain. Our results indicate that simple and rapid OOD detection techniques based on traditional algorithms such as KNN using engineered statistical features outperform sophisticated deep learning techniques on some HAR time series datasets.

## Figures and Tables

**Table 1:** Dataset Characteristics.

Dataset	Ns	Ne	Type	Sensors	fz [Hz]	Time [h]
SPARS	20	7	Shoulder Physiotherapy	Wrist 6-axis IMU	50	3.4
SPARS9x ^a^	20	6	Shoulder Physiotherapy	Wrist 9-axis IMU	50	95.4
MHealth ^b^	10	12	General Fitness	Wrist 9-axis IMU	50	1.9

Ns, number of subjects; Ne, number of exercises; f_s_, sampling frequency. ^a^ Four isometric exercises are included in the SPARS9x dataset, but these are excluded for this study. ^b^ Only the data corresponding to the wrist-IMU of MHEALTH are used.

**Table 2:** In-Distribution Classification Results.

	Accuracy % (Confidence Interval)														
	MHEALTH Segment Length					SPARS Segment Length					SPARS9x Segment Length				
Method	2.0s	4.0s	6. 0s	8.0s	10.0s	2.0s	4.0s	6. 0s	8.0s	10.0s	2.0s	4.0s	6. 0s	8.0s	10.0s
KNN	88.0 (3.0)	87.7 (2.3)	86.5 (2.2)	87.7 (2.4)	87.1 (1.5)	81.0 (1.7)	82.3 (2.6)	82.9 (0.83)	81.4 (2.8)	82.2 (1.8)	96.4 (0.67)	97.6 (0.78)	97.8 (0.71)	97.7 (0.80)	97.7 (0.77)
KNN (CRNN Deep Features)	95.2 (1.4)	94.1 (2.3)	94.8 (1.3)	**96.0 (1.1)**	92.7 (1.4)	87.7 (0.86)	67.5 (4.7)	81.2 (1.0)	78.7 (1.7)	75.6 (1.2)	97.6 (0.41)	96.6 (0.76)	96.8 (0.92)	95.5 (0.57)	94.8 (0.61)
KNN (FCN Deep Features)	93.1 (0.8)	93.7 (2.3)	93.3 (1.7)	92.8 (1.7)	93.1 (1.8)	89.9 (0.98)	92.5 (1.2)	92.2 (1.1)	**93.1 (1.4)**	92.1 (1.5)	98.9 (0.19)	99.8 (0.12)	99.7 (0.14)	**99.9 (0.066)**	99.4 (0.43)
CRNN	94.8 (1.3)	93.8 (2.3)	94.6 (1.4)	95.9 (1.2)	90.0 (2.2)	87.6 (0.87)	67.4 (5.1)	80.8 (1.3)	76.2 (1.7)	72.9 (0.76)	97.7 (0.46)	96.6 (0.76)	96.7 (0.94)	95.4 (0.53)	94.7 (0.64)
FCN	94.2 (1.6)	94.8 (1.6)	93.0 (0.79)	93.5 (1.4)	95.3 (1.3)	87.9 (1.4)	86.4 (1.6)	87.1 (1.6)	87.9 (1.2)	88.0 (2.4)	98.5 (0.38)	99.7 (0.19)	99.6 (0.20)	**99.9 (0.066)**	98.1 (1.7)

**Table 3:** Out-of-Distribution Detection Results.

	AUROC (Confidence Interval)														
	MHEALTH Segment Length					SPARS Segment Length					SPARS9x Segment Length				
Method	2.0s	4.0s	6. 0s	8.0s	10.0s	2.0s	4.0s	6. 0s	8.0s	10.0s	2.0s	4.0s	6. 0s	8.0s	10.0s
**Traditional Methods – Engineered Statistical Features**															
KNN	0.903 (0.026)	0.902 (0.019)	**0.905 (0.018)**	0.904 (0.017)	0.898 (0.014)	0.865 (0.027)	0.912 (0.021)	0.920 (0.019)	0.927 (0.018)	0.934 (0.017)	0.918 (0.0028)	0.963 (0.0033)	0.975 (0.0029)	0.980 (0.0019)	**0.982 (0.0021)**
Kmeans	0.881 (0.020)	0.887 (0.015)	0.886 (0.010)	0.884 (0.014)	0.878 (0.0064)	0.842 (0.021)	0.892 (0.024)	0.903 (0.019)	0.913 (0.018)	0.917 (0.017)	0.872 (0.011)	0.937 (0.0059)	0.955 (0.0046)	0.964 (0.0043)	0.969 (0.0042)
OCSVM	0.796 (0.019)	0.796 (0.027)	0.784 (0.022)	0.776 (0.023)	0.759 (0.015)	0.802 (0.027)	0.863 (0.029)	0.871 (0.028)	0.883 (0.027)	0.887 (0.027)	0.804 (0.017)	0.896 (0.010)	0.927 (0.0080)	0.940 (0.00079)	0.949 (0.0074)
**KNN – Deep Feature Embedding**														
CRNN Features	0.852 (0.017)	0.839 (0.024)	0.791 (0.055)	0.839 (0.031)	0.837 (0.047)	0.903 (0.023)	0.858 (0.020)	0.859 (0.029)	0.829 (0.052)	0.877 (0.029)	0.754 (0.017)	0.819 (0.0074)	0.794 (0.011)	0.788 (0.0053)	0.774 (0.020)
FCN Features	0.854 (0.023)	0.883 (0.024)	0.874 (0.014)	0.877 (0.017)	0.891 (0.028)	0.969 (0.0080)	0.976 (0.0073)	0.971 (0.0064)	0.974 (0.0082)	**0.978 (0.0058)**	0.921 (0.011)	0.960 (0.0053)	0.967 (0.0076)	0.965 (0.0062)	0.965 (0.0060)
**CRNN Core Model**															
Softmax Threshold	0.611 (0.048)	0.609 (0.047)	0.553 (0.057)	0.656 (0.038)	0.578 (0.068)	0.777 (0.036)	0.819 (0.014)	0.840 (0.020)	0.788 (0.026)	0.839 (0.024)	0.700 (0.016)	0.718 (0.013)	0.680 (0.014)	0.669 (0.025)	0.634 (0.031)
Entropy Regularization	0.736 (0.015)	0.731 (0.032)	0.680 (0.064)	0.705 (0.035)	0.644 (0.067)	0.911 (0.0080)	0.917 (0.0091)	0.928 (0.012)	0.903 (0.019)	0.930 (0.0087)	0.691 (0.021)	0.689 (0.024)	0.708 (0.015)	0.690 (0.023)	0.636 (0.039)
ODIN	0.633 (0.036)	0.664 (0.050)	0.597 (0.060)	0.546 (0.082)	0.512 (0.036)	0.860 (0.0086)	0.860 (0.020)	0.846 (0.029)	0.855 (0.010)	0.815 (0.032)	0.692 (0.017)	0.698 (0.017)	0.636 (0.037)	0.668 (0.029)	0.595 (0.030)
OpenMax	0.661 (0.050)	0.614 (0.076)	0.693 (0.053)	0.707 (0.055)	0.589 (0.059)	0.840 (0.034)	0.863 (0.0036)	0.873 (0.024)	0.851 (0.037)	0.838 (0.036)	0.776 (0.019)	0.694 (0.058)	0.689 (0.035)	0.682 (0.066)	0.758 (0.036)
**FCN Core Model**															
Softmax Threshold	0.731 (0.016)	0.622 (0.039)	0.680 (0.055)	0.600 (0.031)	0.597 (0.017)	0.779 (0.024)	0.812 (0.018)	0.783 (0.042)	0.799 (0.034)	0.795 (0.034)	0.756 (0.014)	0.752 (0.012)	0.759 (0.017)	0.764 (0.0082)	0.771 (0.0058)
Entropy Regularization	0.752 (0.018)	0.637 (0.039)	0.666 (0.062)	0.610 (0.039)	0.672 (0.023)	0.792 (0.027)	0.815 (0.024)	0.791 (0.037)	0.781 (0.037)	0.807 (0.034)	0.747 (0.015)	0.752 (0.011)	0.762 (0.021)	0.743 (0.017)	0.772 (0.014)
ODIN	0.699 (0.022)	0.601 (0.030)	0.649 (0.026)	0.645 (0.035)	0.673 (0.046)	0.820 (0.022)	0.816 (0.023)	0.826 (0.031)	0.849 (0.020)	0.821 (0.016)	0.743 (0.017)	0.752 (0.012)	0.747 (0.025)	0.735 (0.020)	0.749 (0.0095)
OpenMax	0.794 (0.036)	0.645 (0.043)	0.734 (0.054)	0.714 (0.069)	0.708 (0.014)	0.845 (0.021)	0.856 (0.013)	0.855 (0.017)	0.875 (0.021)	0.850 (0.030)	0.910 (0.011)	0.897 (0.020)	0.916 (0.011)	0.915 (0.020)	0.922 (0.019)

**Table 4:** Out-of-Distribution Prediction Time.

Method	Prediction Time (s)		
	MHEALTH	SPAR	SPARS9x
**Traditional Methods—Engineered Statistical Features**			
KNN	0.66 (0.002)	0.37 (0.005)	2.47 (0.03)
Kmeans	0.47 (0.001)	0.32 (0.007)	1.46 (0.02)
OCSVM	0.52 (0.002)	0.35 (0.005)	2.11 (0.03)
**KNN—Deep Feature Embedding**			
CRNN Features	0.10 (0.0008)	0.15 (0.006)	0.93 (0.05)
FCN Features	0.16 (0.0009)	0.30 (0.01)	2.00 (0.03)
**CRNN Core Model**			
Softmax Threshold	0.066 (0.002)	**0.082 (0.004)**	**0.41 (0.005)**
Entropy Regularization	**0.059 (0.0003)**	0.084 (0.004)	**0.41 (0.005)**
ODIN	0.22 (0.0003)	0.34 (0.02)	1.66 (0.02)
OpenMax	3.39 (0.08)	0.93 (0.02)	5.41 (0.06)
**FCN Core Model**			
Softmax Threshold	0.099 (0.0004)	0.22 (0.007)	1.09 (0.01)
Entropy Regularization	0.14 (0.0008)	0.21 (0.003)	0.84 (0.03)
ODIN	0.43 (0.001)	0.69 (0.002)	3.16 (0.10)
OpenMax	3.64 (0.09)	1.04 (0.02)	6.04 (0.06)

**Table 5:** Training Time.

Model	Training Time (s)		
	MHEALTH	SPAR	SPARS9x
KNN	3.08 (0.009)	4.69 (0.02)	5.58 (0.02)
Kmeans	1.98 (0.06)	3.03 (0.03)	3.50 (0.07)
OCSVM	2.43 (0.06)	4.40 (0.03)	5.74 (0.05)
CRNN core	81.2 (0.05)	124 (0.7)	144 (1.0)
FCN core	164 (6.0)	261 (7.4)	317 (1.0)

## Data Availability

The SPARS9x dataset presented in this study is publicly available in the open access repository “Shoulder Physiotherapy Activity Recognition 9-Axis Dataset” DOI: 10.21227/cx5v-vw46.
